# Spectacular rediscovery of the original prints of radiographs Roentgen sent to Lorentz in 1896

**DOI:** 10.1186/s13244-020-00846-x

**Published:** 2020-03-03

**Authors:** Frans W. Zonneveld

**Affiliations:** Historical Committee of the Dutch Radiological Society, Formerly: Department of Radiology, Utrecht University Medical Center, Oranjestraat 35, 5091 BK Middelbeers, The Netherlands

**Keywords:** First radiographs, Discovery of X-rays, W.C. Roentgen’s experiments, H.A. Lorentz, Teylers Museum

## Abstract

**Background:**

Ninety years after the Dutch theoretical physicist H.A. Lorentz died, detailed investigation of his scientific heritage yielded the set of nine original prints of radiographs that W.C. Roentgen made during his experiments and had sent him, among half a dozen other scientists, on January 1^st^, 1896.

**Main text:**

Through communications with different experts and literature research, the author describes these nine prints and how they relate to the first publication Roentgen wrote about his discovery of the X-rays.

**Conclusions:**

The combination of Roentgen’s first publication on his X-ray discovery and the nine radiographs provides insight as to which aspects of the discovery were considered important by Roentgen and how he carried out the experiments to be able to describe these aspects.

## Key points

- W.C. Roentgen did his experiments after his discovery of a ‘new kind of rays’ in a very analytic and orderly manner.
- W.C. Roentgen has sent the prints of radiographs related to his publication, combined with the reprint, to a select group of about a dozen internationally renowned scientists while he sent the reprint as such, without the illustrations, to some 80 other scientists.
- Only two presumably full sets of the original radiographs are now known in the world.

## Introduction

**Fig. 1:**
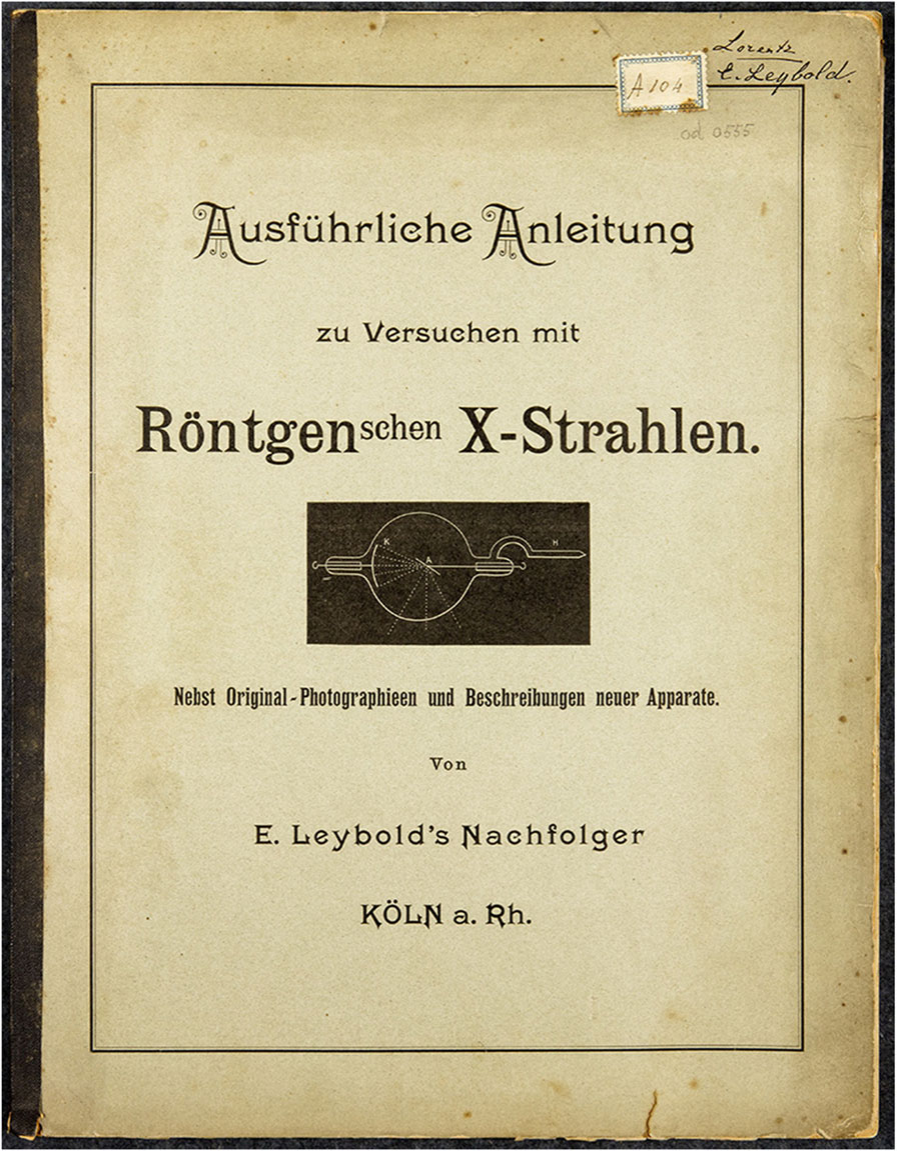
Catalogue (1896) of X-ray equipment produced by the Leybold Company (Cologne, Germany) including a guide on how to take radiographs using this equipment

**Fig. 2:**
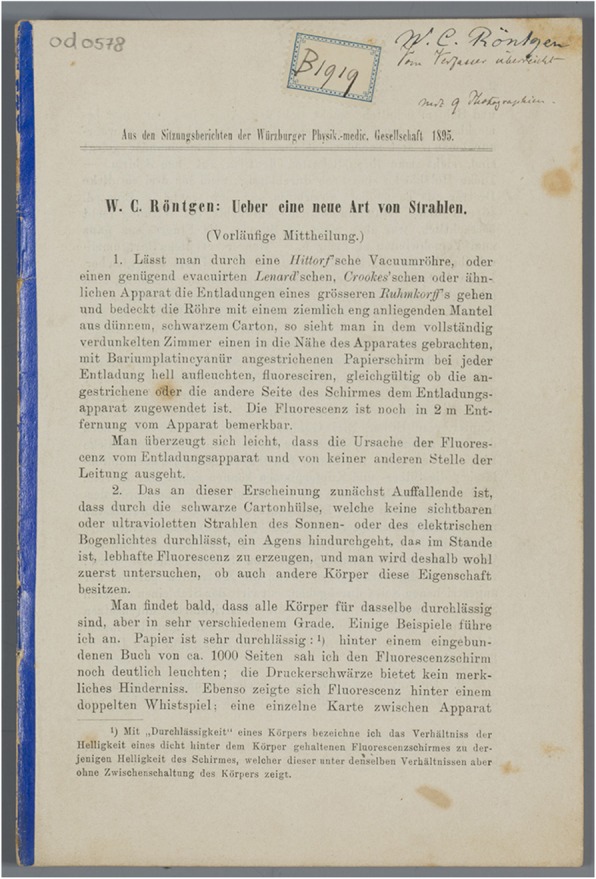
Reprint of the first publication by Roentgen on his discovery of a new kind of rays. This is the reprint with the blue rim on its spine that was sent to Lorentz

Since Hendrik Antoon Lorentz^1^ [2] passed away (February 4th, 1928), part of his scientific heritage^2^ has been kept at Teylers Museum^3^, Haarlem, The Netherlands, where Lorentz was the director (since 1909) of the Physics Laboratory of the Teylers Foundation^4^. As this heritage consisted of many books, papers and thousands of reprints, it had never been studied in detail. In the course of 2018, museum workers embarked on this enormous undertaking. Between the pages of an 1896 catalogue^5^ (Fig. 1) of the Leybold Company (Cologne, Germany), they encountered, near the letter L (personal communication with Mrs. T. van der Spek, head curator of the scientific collections and head of the science department at Teylers Museum), a folder containing 13 prints of radiographs. Nine of these radiographs presumably formed the original set that Roentgen had sent to about half a dozen of his scientific colleagues on January 1st, 1896. The other four prints of radiographs did not originate from Roentgen and were of later dates. Two of these prints could be traced back to an X-ray demonstration by a local high school teacher in the city of Tilburg (The Netherlands) on March 18th 1896. Near the letter R in the catalogue, the original reprint [3] (Fig. 2) of Roentgen’s first publication, which he had sent along with the prints of radiographs, was found. It contained Roentgen’s handwritten note: ‘W.C. Röntgen. Vom Verfasser überreicht mit 9 Photographien’ (W.C. Roentgen. Presented by the author with nine photographs^6^).

## Can we discover to whom Roentgen had sent the prints of radiographs and reprints?

Most of the people in Roentgen’s network only received the reprint^7^. The first batch of these had a blue rim on its spine, and the remaining batch had a yellow rim. We only know six names of those who received both the reprint and the set of radiographs in early 1896. They are Franz-Serafin Exner (1849–1926) in Vienna, William Thomson (Lord Kelvin) (1824–1907) in Glasgow, Emil Gabriel Warburg^8^ (1846–1931) in Berlin, Franz Arthur Friedrich Schuster (1851–1934) in Manchester, Henri Poincaré (1854–1912) in Paris and Hendrik Antoon Lorentz (1853–1928) in Leiden. Later, he sent a similar material to a number of German colleagues. Of them, we know the following names: Otto Richard Lummer (1860–1925) in Berlin, Friedrich Wilhelm Georg Kohlrausch^9^ (1840–1910) in Strasbourg and Carl August Voller (1842–1920) in Hamburg. Until now, the only presumably complete set preserved is the one that was sent to Schuster. It was donated by his daughter to the Wellcome Collection in London in the 1960s. Roentgen was befriended with Ludwig Luis Albert Zehnder^10^ (1854–1949) in Freiburg but sent him the reprint only. Via his frequent correspondence with Roentgen [4], Zehnder must have received several prints of radiographs which have been preserved at the German Radiology Museum in Remscheid-Lennep (personal communication with Dr. U. Busch, head curator of the German Radiology Museum in Remscheid-Lennep) or he could have received them from others after Roentgens’s death (personal communication with G.J.E. Rosenbusch).

## The set of nine prints of radiographs

**Fig. 3:**
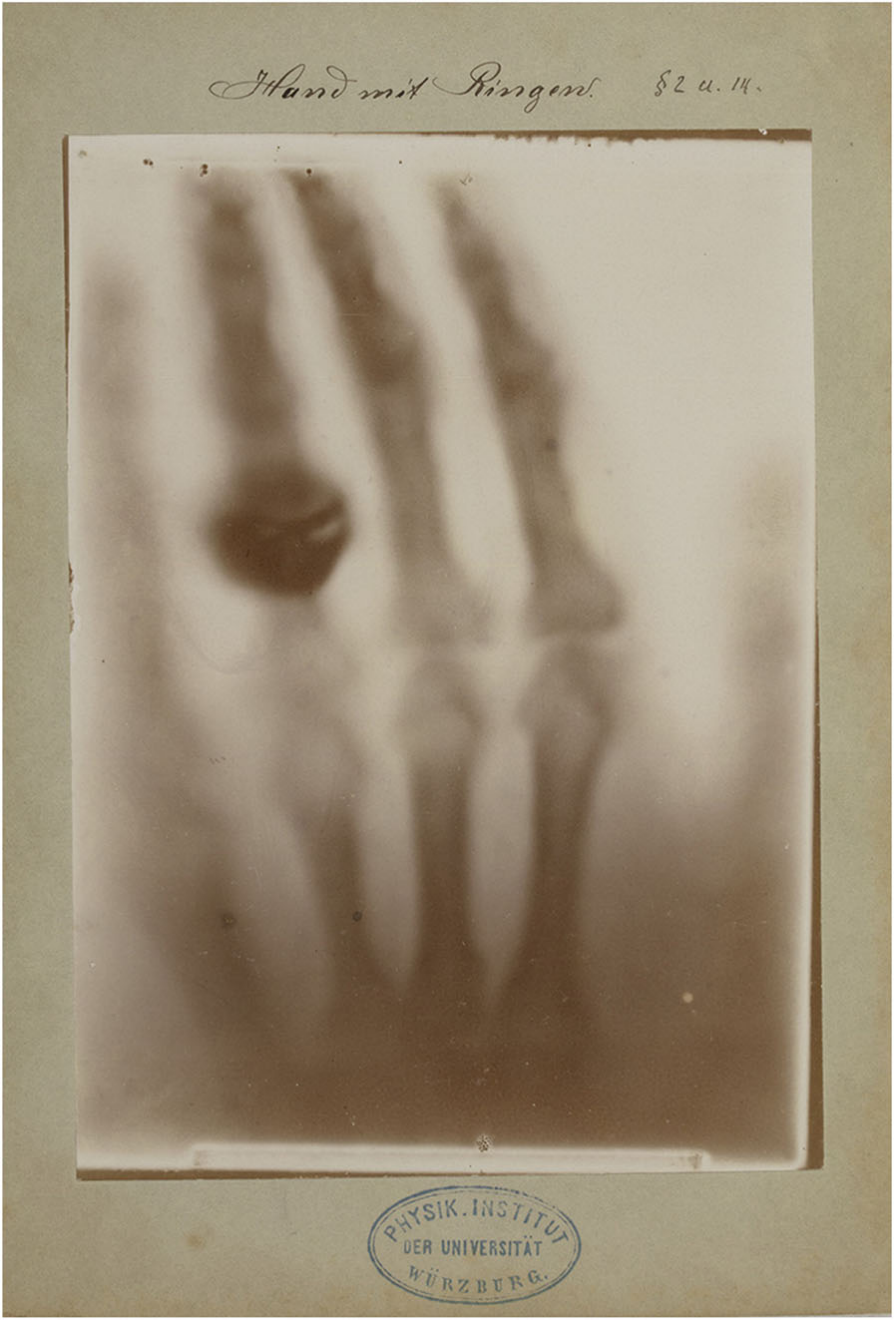
The most impressive radiographic image is the one taken on December 22nd, 1895 of Bertha’s hand as it is the only image which shows the importance of X-rays for medicine as it is the first anatomical radiograph. It is therefore seen as the beginning of radiography. For *Time Magazine*, it was the reason to nominate it as one of the 100 most influential photographs ever. The other eight radiographs are of a technical nature. Manually written legend: ‘Hand mit Ringen’ (Hand with rings). Paragraph referral: § 2 and § 14

The nine images could be considered as illustrations to the first publication although they were not included in it. Therefore, someone^11^ wrote the numbers of the corresponding paragraphs on each cardboard frame. For example, in Fig. 3, we read: ‘§ 2 u. 14’ which stands for ‘paragraphs 2 and 14’. There were 17 paragraphs in total. Captions of what the radiographs showed were added as well.

We will discuss these nine prints of radiographs in brief in the figure captions (Figs. 3, 4, 5, 6, 7, 8, 9 and 10)

**Fig. 4:**
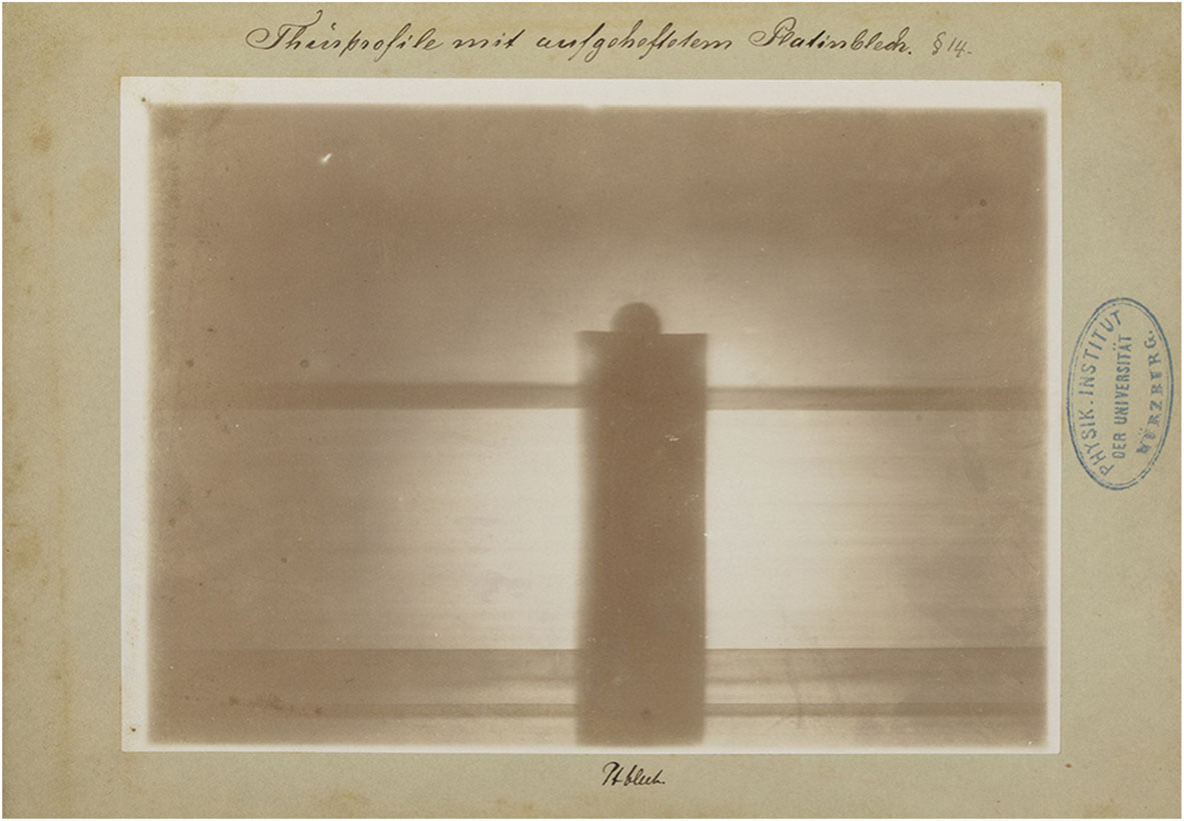
The doorpost of Roentgen’s lab upon which he attached a thin sheet of Platinum. It shows that the X-ray attenuation of the thin plate of platinum is much larger than that of the thick wooden doorpost. Manually written legend: ‘Thürprofile mit aufgeheftetem Platinblech’ (Doorpost with attached thin plate of Platinum). Paragraph referral: § 14

**Fig. 5:**
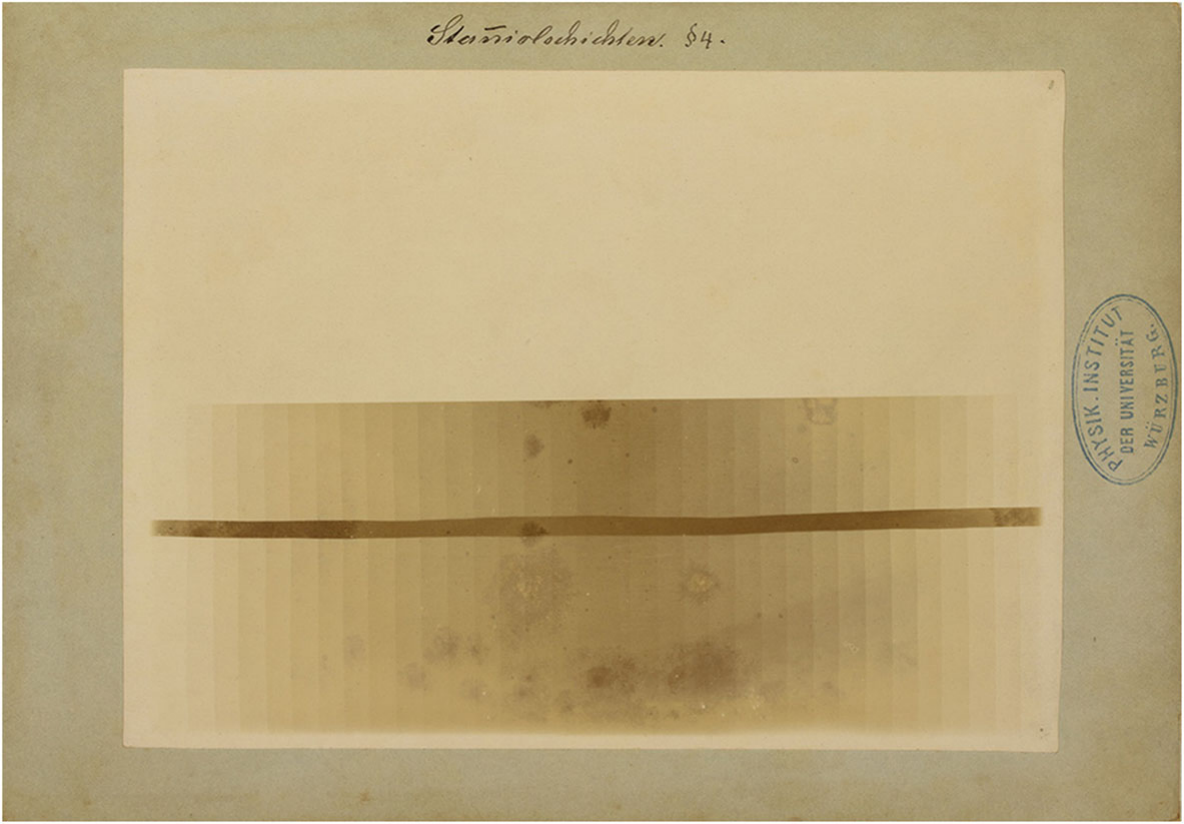
Stacks of tin foil with various thicknesses. This image shows that the thicker the stack, the less X-radiation passes through it. Manually written legend: ‘Stanniolschichten’ (Layers of tin foil). Paragraph referral: § 4

**Fig. 6:**
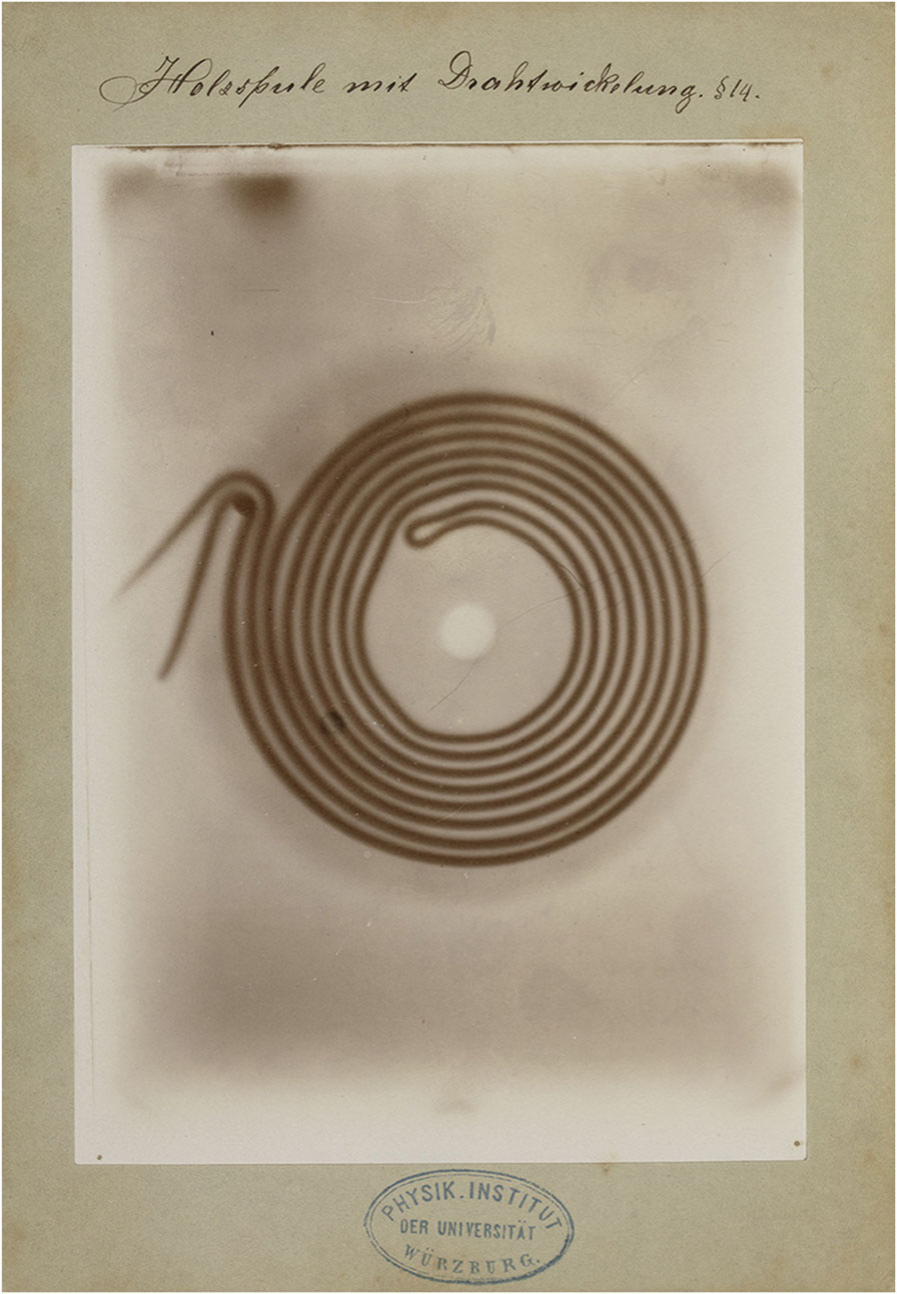
A wooden take-up spool with wire. This image shows that the wood of the spool is practically transparent to the X-rays while the wire is not. Manually written legend: ‘Holzspule mit Drahtwickelung’ (Wooden take-up reel with wire). Paragraph referral: § 14

**Fig. 7:**
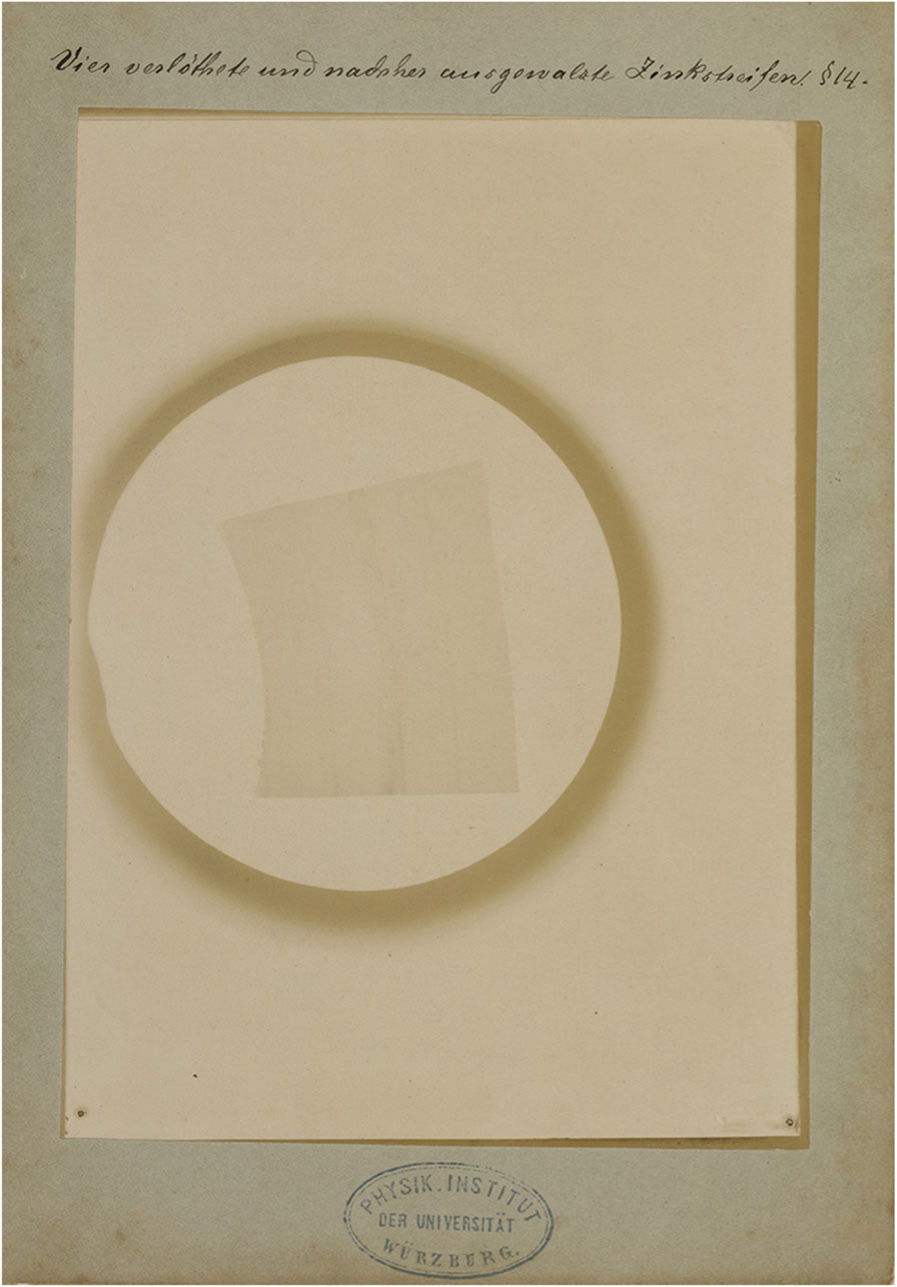
Strips of zinc that have been soldered together and subsequently rolled out to a uniform thickness. This image shows faint shadows where the soldering took place which shows that the difference in atomic number and density between the zinc and the soldering (consisting of tin and lead) is enough to show up on the radiographic image although the thickness is the same everywhere. Manually written legend: ‘Vier verlöthete und nachher ausgewalste Zinkstreifen” (four zinc strips soldered together and afterwards rolled out into one plate). Paragraph referral: § 14

**Fig. 8:**
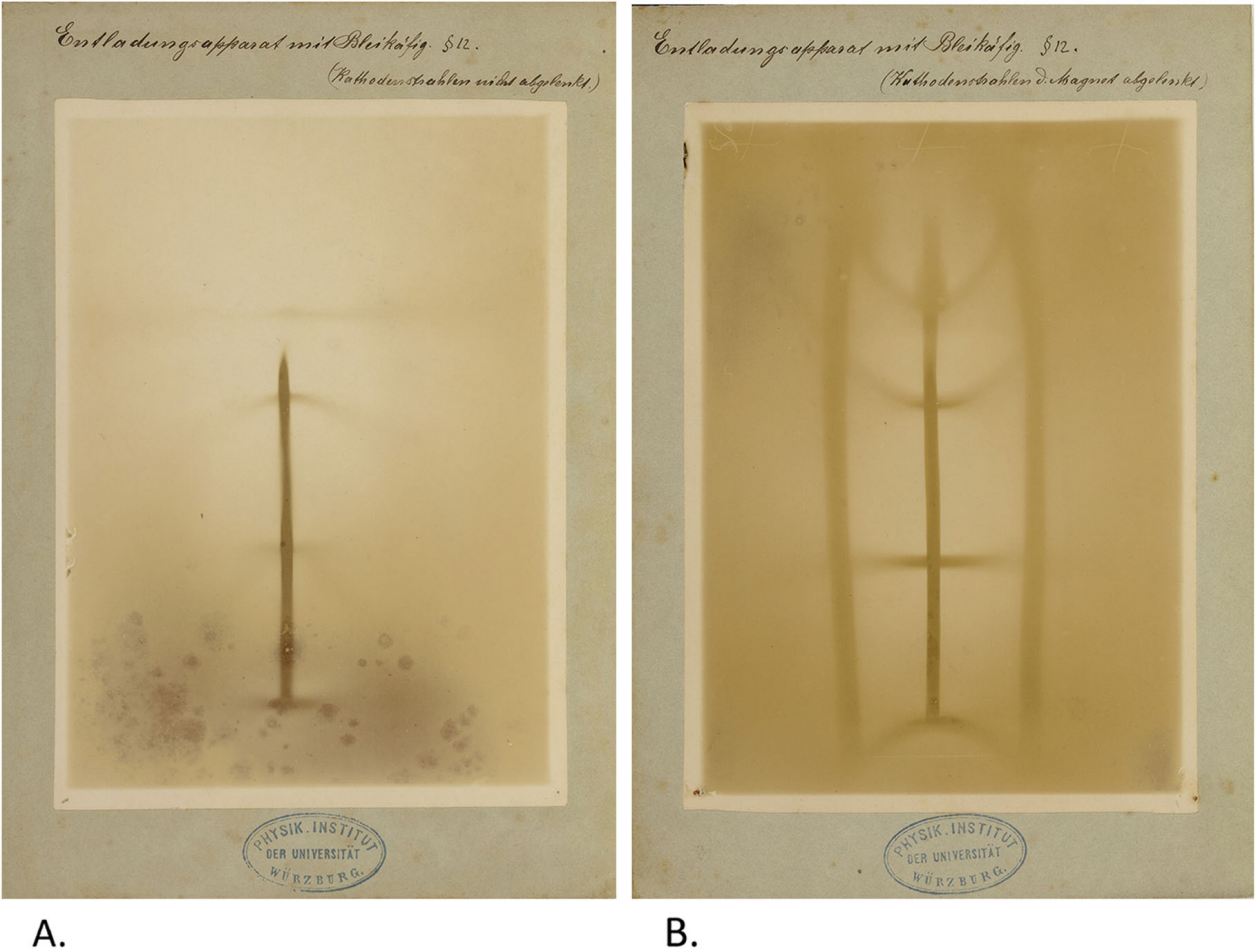
**a**, **b** Two radiographic images of a leaden cage that surrounds the gas discharge tube. The difference between them is that the second image was taken while the cathode rays were deflected using a magnet while in the first image the magnet was absent. Between the two radiographs, the shadow of the leaden cage moved. Roentgen concluded from this that the X-rays had to emanate from the point on the glass wall of the discharge tube where the cathode rays impinged and this point had moved as a result of the deflection of these cathode rays. It can be noticed that the magnet pulls the focal spot downward, resulting in the central ray to hit the center of the cage (shadow of the ribs at the top of the cage bend upward and the brightest part of the background moves downward) while, at the same time, the focal spot becomes smaller (image becomes sharper). Respective manually written legends: ‘Entladungsapparat mit Bleikäfig (Kathodenstrahlen nicht abgelenkt)’(Gas discharge tube with leaden cage (cathode rays not deflected)) and ‘Entladungsapparat mit Bleikäfig (Kathodenstrahlen d[*urch*] (The word ‘durch’ was completed by the author) Magnet abgelenkt)’ (Gas discharge tube with leaden cage (cathode rays deflected by a magnet)). Paragraph referral: § 12 (in both radiographs)

**Fig. 9:**
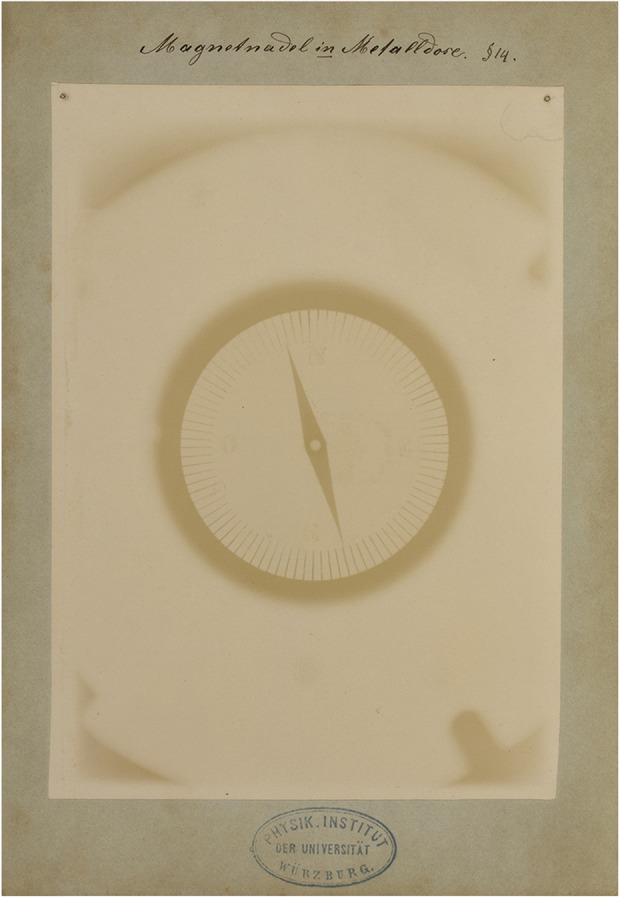
A compass in a closed metal box. This image shows that, if the metal of the box is not too thick, the X-rays will pass and thus visualize the compass needle. Manually written legend: ‘Magnetnadel in Metalldose’ (compass needle inside a metal box). Paragraph referral: § 14

**Fig. 10:**
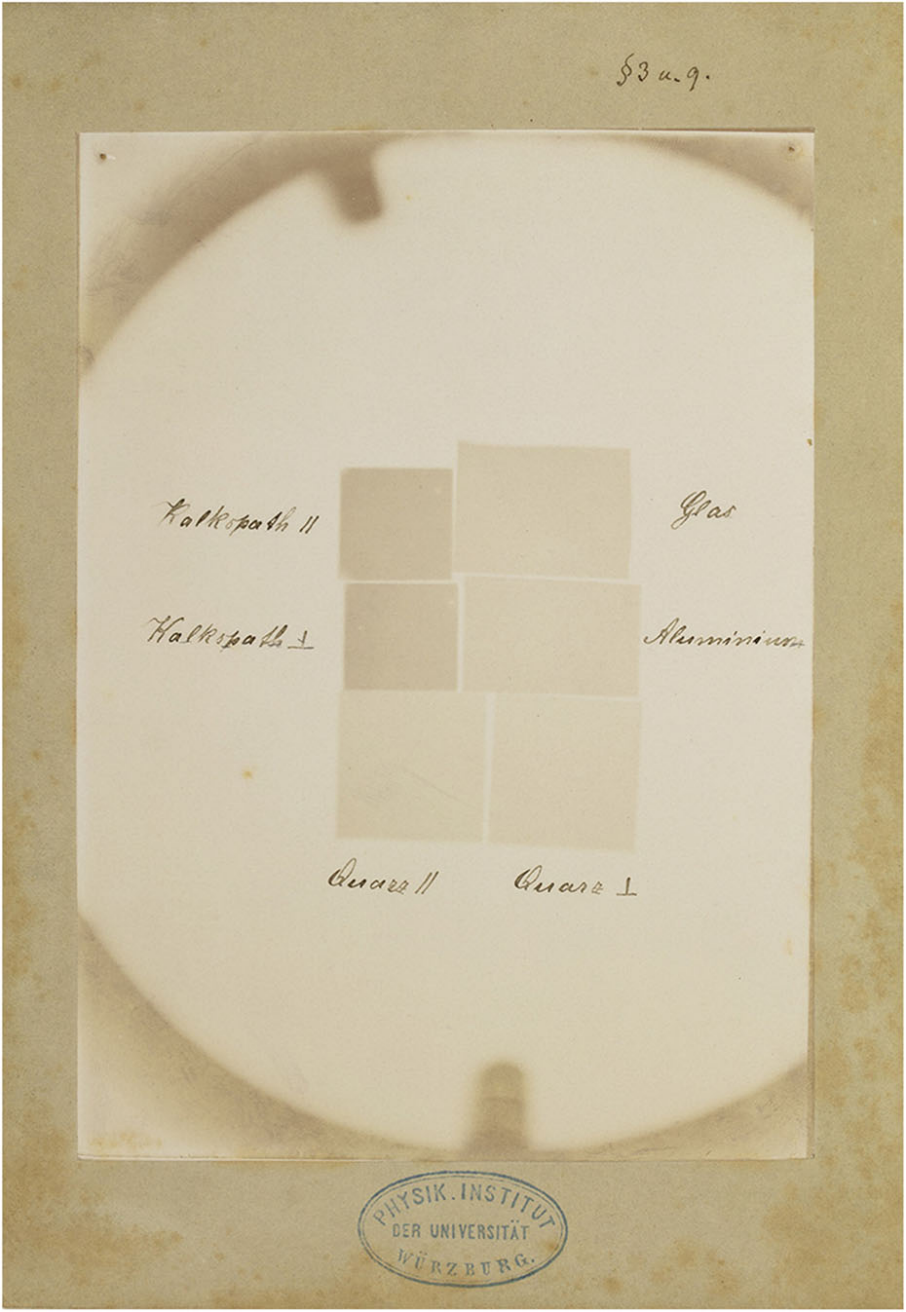
Roentgen was aware of the existence of optical birefringence (a crystal is said to be optically birefringent, or double refracting, if its refractive index depends on the polarization and propagation direction of the light with respect to the crystal orientation; a necessary condition is that the crystal is anisotropic; many such birefringent crystals, e.g. calcite and quartz, have a single axis of symmetry). Roentgen ‘s experiments with prisms to observe refraction of X- rays failed, because, as later was measured, the index of refraction of X-rays is many orders of magnitude smaller than that of visible light. Therefore, the likelihood of observing double refraction in birefringent materials would also have been negligible (even if he had been able to realize the experimental conditions as used for visual light experiments, which he wasn’t). Nevertheless he may have wondered if for X-rays the transmission (and not the refraction) was dependent on the crystal orientation with respect to the beam direction. This would explain why he compared the transmission parallel (//) to the symmetry axis of the birefringent crystals ‘Kalkspath’ and ‘Quarz’ with that in a direction perpendicular (⊥) to it. As we now know, the attenuation (and thus the transmission) of X-rays is determined by the atomic number Z, the density and the thickness of the attenuating material, so here no effect was to be expected either. Manually written legend on the photographic paper: ‘Glas, Aluminium, Quarz ⊥, Quarz //, Kalkspath ⊥, Kalkspath //’ (glass, aluminum, quartz ⊥, quartz //, calcite ⊥, calcite //). Paragraph referral: § 3 and § 9)

## Reactions to the first mailing by Roentgen

As Roentgen had predicted [5], the reactions came soon. Lord Kelvin wrote a formal thank-you-note on January 6th after having only seen the prints of radiographs, but, after reading the paper, he wrote a second letter on January 17th to express his astonishment [5].

Lorentz was very impressed by Roentgen’s discovery, especially because it fitted perfectly with his own ideas. Lorentz was of the English school which believed that cathode rays were of a corpuscular character. We can find this vision in the article that Lorentz was asked to write for the Dutch journal ‘De Gids’ which was published on February 19th, 1896 [6]. Lenard, on the contrary, thought that cathode rays were a distortion of the ether and thus had a wave character. Thompson, in 1897, showed that cathode rays were electrons, thus particles, but, in the end, the supporters of the wave theory had it partly their way at the introduction of the quantum theory: electrons have also wave character.

The radiographs which Exner received were instrumental in spreading the news about Roentgen’s discovery as Exner showed them to the young physicist Ernst Lechner (1856–1926) who told his father (editor of the Viennese newspaper ‘Die Presse’), who published the news on January 5th, 1896 [5].

## Reactions to the rediscovery of the prints of radiographs

The rediscovery of the original prints of radiographs resulted in multiple reactions in the Dutch press. The Teylers Museum reacted by putting Roentgens prints of radiographs (as well as the reprint and the other prints of radiographs) on display for a month.

## Comparison with other sets of prints of radiographs Roentgen sent to his colleagues

The prints of radiographs of the set sent to Schuster are reproduced on the website of the Wellcome Foundation^12^. The only difference with Lorentz’ set is that the corners of the frame of the radiograph of Bertha’s hand in Schuster’s set are seriously damaged. The remainder of the set is identical to the one sent to Lorentz. Of the prints of radiographs in possession of Zehnder, five have been preserved in the German Radiology Museum in Remscheid-Lennep, among which is the famous radiograph of the hand of Roentgen’s wife Bertha. The four that are missing are our Figs. 4, 6, 7 and 10. However, there is one additional X-ray photograph which was not present in the Lorentz and Schuster sets and which is also clearly an illustration of Roentgen’s first publication. This is an attempt to refract the X-rays with 30 degree vulcanite and aluminum prisms. It refers to paragraph 7 (personal communication with Dr. U. Busch, head curator of the German Radiology Museum in Remscheid-Lennep). Roentgen states that he could not demonstrate that the X-rays were refracted but if they did it would be to a minimal degree. It is not known why this image was sent to Zehnder^13^ and not to Lorentz and Schuster. Zehnder also received a radiograph of Roentgen’s hunting rifle, which was made after Roentgen’s presentation to the German emperor on January 13th, 1896; the reason being that during this visit in Berlin, the general staff had asked Roentgen whether it would be possible to use the X-rays to examine materials, especially those of rifles (personal communication with Dr. U. Busch, head curator of the German Radiology Museum in Remscheid-Lennep). Roentgen made many notes on this photograph explaining what can be seen^14^. It is, obviously, not an illustration of the publication but falls into the same category as the image of the hand (Fig. 3), the doorpost (Fig. 4), the wooden spool (Fig. 6) and the wooden box with a set of weights [7] which are specifically mentioned in paragraph 14 of the publication. It remains unclear how Zehnder received the prints of radiographs that are now in the Remscheid-Lennep Museum and requires further investigation. We do know [5] that Zehnder had some of the missing prints of radiographs temporarily in his possession as he borrowed them from Roentgen for a presentation on February 15th, 1896 for the ‘Naturforschende Gesellschaft’ (Society for Nature Research) in Freiburg. These were the wooden spool (Fig. 6), the zinc plate (Fig. 7), the wooden box with a set of weights [7] and a radiograph of a hand made by Pernet^15^ in Zurich.

## Data Availability

All figures originate in the Teylers Museum. They are the owner of the copyright.

## Footnotes

1 H.A. Lorentz is one of the greatest theoretical physicists of the Netherlands (Nobel Prize for Physics in 1902 together with Pieter Zeeman). He was professor at Leiden University. Roentgen travelled to Leiden in early 1905 and asked Lorentz to become professor of theoretical physics in Munich [1] but Lorentz turned this proposal down. Lorentz moved to Haarlem in 1912. (https://en.wikipedia.org/wiki/Hendrik_Lorentz Accessed 4 October 2019)

2 Lorentz heritage in Teylers Museum consists of papers and books that he brought with him from Leiden plus everything related to his work as director of the Physics Lab at Teylers Museum. The remainder of his archive is located at the North-Holland Archive (www.noord-hollandsarchief.nl).

3 Teylers Museum is a museum for science and art. Founded in 1778, it is the oldest museum of the Netherlands.

4 Lorentz was also curator of the physics cabinet of Teylers Museum.

5 The catalogue was also a guide on how to make radiographs using the equipment of the Leybold Company.

6 Roentgens mailing actually contained positive photographic prints of his original negative radiographs.

7 Without including the prints of radiographs, Roentgen sent this reprint to some eighty other scientific colleagues. Among them were three other Dutch scientists: J.D. van der Waals (1837–1923) at the University of Amsterdam, C.H.C. Grinwis (1831–1899) at the University of Utrecht, and H. Haga (1852–1936) at the University of Groningen.

8 Predecessor of Roentgen in Strasbourg.

9 Predecessor of Roentgen in Würzburg.

10 Assistant of Roentgen in Strasbourg and Würzburg and a later colleague of Roentgen in Munich. After Roentgens death, he received the letters he had sent to Roentgen, back, except the ones written in the period of the discovery (Personal communication with Gerd J.E. Rosenbusch).

11 The handwriting is clearly different from Roentgens handwriting.

12 https://wellcomecollection.org/works?query=%22Radiograph%20by%20Roentgen%22%20%22Arthur%20Schuster%20Schuster%22 Accessed 11 February 2020

13 The correspondence between Roentgen and Zehnder is archived at the Zentralbibliotek (Central Library) in Zurich, Switzerland. It is possible that somewhere in this correspondence there are referrals to the prints of radiographs that Zehnder received from Roentgen, but it is not possible for this library to perform this research for me (Personal communication with Monica Seidler-Hux, scientific coworker of the manuscript department).

14 Roentgen sent this X-ray photograph also to the German emperor along with three prints of radiographs of 23 mm thick iron plates; according to a concept of the letter to the emperor which dates back to January 12^th^ 1897 (Personal communication with Gerd J.E. Rosenbusch).

15 Jean Pernet (1845-1902) was Professor of Experimental Physics at the ETH Zurich (1890-1902).
